# Cross-reactive mouse monoclonal antibodies raised against the hemagglutinin of A/Shanghai/1/2013 (H7N9) protect against novel H7 virus isolates in the mouse model

**DOI:** 10.1038/s41426-018-0115-0

**Published:** 2018-06-20

**Authors:** Daniel Stadlbauer, Fatima Amanat, Shirin Strohmeier, Raffael Nachbagauer, Florian Krammer

**Affiliations:** 1 Department of Microbiology, Icahn School of Medicine at Mount Sinai, New York, NY USA; 2 Department of Biotechnology, University of Natural Resources and Life Sciences, Vienna, Austria

## Abstract

Influenza viruses remain a major global public health risk. In addition to seasonal influenza viruses, epizootic influenza A H7 subtype viruses of both the Asian and North American lineage are of concern due to their pandemic potential. In China, the simultaneous occurrence of H7N9 zoonotic episodes and seasonal influenza virus epidemics could potentially lead to novel reassortant viruses with the ability to efficiently spread among humans. Recently, the H7N9 virus has evolved into two new lineages, the Pearl River Delta and the Yangtze River Delta clade. This development has also resulted in viruses with a polybasic cleavage site in the hemagglutinin that are highly pathogenic in avian species and have caused human infections. In addition, an outbreak of a highly pathogenic H7N8 strain was reported in the US state of Indiana in 2016. Furthermore, an H7N2 feline virus strain caused an outbreak in cats in an animal shelter in New York City in 2016, resulting in one human zoonotic event. In this study, mouse monoclonal antibodies previously raised against the hemagglutinin of the A/Shanghai/1/2013 (H7N9) virus were tested for their (cross-) reactivity to these novel H7 viruses. Moreover, the functionality of these antibodies was assessed in vitro in hemagglutination inhibition and microneutralization assays. The therapeutic and prophylactic efficacy of the broadly reactive antibodies against novel H7 viruses was determined in vivo in mouse passive transfer-viral challenge experiments. Our results provide data about the conservation of critical H7 epitopes and could inform the selection of pre-pandemic H7 vaccine strains.

## Introduction

Influenza viruses are a public health concern on a global scale^1^. Annually, influenza viruses infect millions of people worldwide resulting in 290,000 to 650,000 influenza-related deaths^2^. Besides globally circulating seasonal influenza strains of the H1N1 subtype, H3N2 subtype, or influenza B strains, avian influenza viruses of the H7 subtype can result in zoonotic infections^3^. In 2017, the fifth wave of a zoonotic H7N9 epidemic emerged in China, resulting in higher numbers of laboratory-confirmed human infections (over 1500) than in previous years, coupled with a high case fatality rate (almost 40%)^4^. While these viruses have not yet gained the capability of sustained human-to-human transmission, they do pose a pandemic risk if the avian virus were to adapt to humans or undergo reassortment with seasonal viruses^5,6^. Human infections with highly pathogenic avian influenza (HPAI) H7N9 viruses with polybasic cleavage sites in the hemagglutinin (HA) have been reported during the most recent epidemic^6^. These HPAI H7N9 virus isolates contained dual receptor binding properties, allowing them to bind to α2,6-linked sialic acid receptors (prevalent in the human upper airways) as well as α2,3-linked sialic acid receptors (prevalent in many avian species)^7^. Additionally, during the 2016–2017 Northern Hemisphere winter season, the A/H7N9 virus evolved and clustered into antigenically distinct lineages^7,8^ the Yangtze River Delta (YRD) lineage and Pearl River Delta (PRD) lineage. When tested against ferret antisera, it was shown that these two lineages did not match H7 stockpiled vaccines well^9^. Outside Mainland China, a highly pathogenic avian H7N8 virus was isolated from commercial turkeys in the US state of Indiana in 2016, causing severe systemic disease and high mortality in these animals^10,11^. Additionally, in New York City, an outbreak of an H7N2 virus in cats in an animal shelter led to public health concerns at the end of 2016. The feline virus caused one known human zoonotic event by infecting a human healthcare worker, who subsequently experienced influenza-like illness^12^.

Humans are immunologically naive to subtype H7 viruses^13^. If zoonotic H7 viruses from animal reservoirs were to adapt to humans through mutations, H7 viruses could gain pandemic potential^14,15^. Vaccination regimens to protect against H7 viruses often only elicit low levels of hemagglutination inhibiting antibody titers and require further development^16–21^. However, the hemagglutination inhibition (HI) assay may not be sufficient to measure the full extent of the antibody response against H7 viruses^19,22,23^. Antibodies that target other regions of the HA, such as the membrane proximal stalk domain, can contribute to protection by mechanisms other than HI, but can only be detected in other types of assays^24–26^.

We have previously generated a set of four murine monoclonal antibodies (mAbs) against the HA of the A/Shanghai/1/2013 H7N9 virus^27^. The panel includes two HI-active and neutralizing mouse mAbs, as well as two non-HI-active and non-neutralizing mouse mAbs which have all been shown to be protective against H7N9 challenge in vivo. Here we tested their (cross-) reactivity and in vitro and in vivo functionality against the newly emerged Eurasian and American lineage H7 viruses described above.

## Results

### Mouse mAbs bind to the HA of novel H7 virus isolates of the Eurasian and North American lineages

**Fig. 1:**
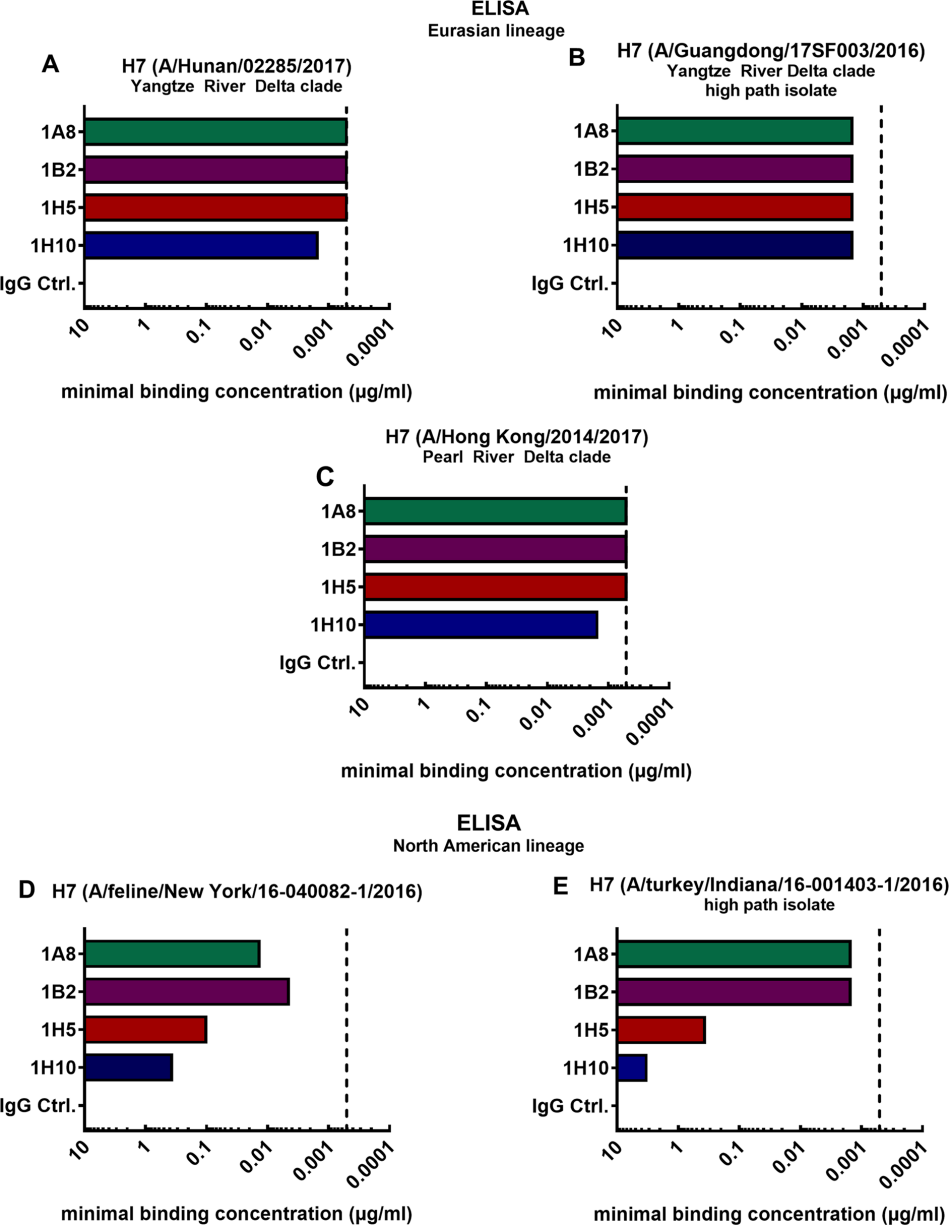
Reactivity of broadly reactive mouse mAbs to HAs of novel H7 virus isolates as measured by ELISA. Minimal binding concentrations of mAbs 1A8, 1B2, 1H5, 1H10 and negative IgG control binding to recombinant H7 HAs of Eurasian lineage virus isolates A/Hunan/02285/2017 (**a**), A/Hong Kong/2014/2017 (**b**), and A/Guangdong/17SF003/2016 (**c**). Minimal binding concentrations of mAbs binding to the H7 HAs of North American lineage HAs of virus isolates A/feline/New York/16-040082/2016 (**d**) and A/turkey/Indiana/16-001403-1/2016 (**e**). The vertical dashed line represents the minimal binding concentration of all four mAbs to H7 HA of A/Anhui/1/2013, which is antigenically similar to the strain they were raised to

The minimal binding concentrations of four broadly reactive mAbs raised against the H7 HA of the A/Shanghai/1/2013 (Shanghai) virus strain were assessed using enzyme-linked immunosorbent assays (ELISAs). The mAbs 1A8, 1B2, 1H5, and 1H10 have been previously generated in our laboratory using hybridoma technology and have been described in detail^27^. It was shown that mAbs 1A8 and 1B2 bind to a wide range of H7 HAs of both the Eurasian and North American lineage. Antibodies 1H5 and 1H10 showed strong binding to Eurasian lineage H7 HAs but displayed weak binding to North American lineage HAs. In this study, we tested binding of these mAbs to H7 HAs of emerging viruses from both lineages (Fig. 1). Here, we detected the minimal binding concentrations performing ELISAs using the recombinantly expressed HA of novel H7 virus strains of the Asian PRD and YRD clade (Fig. 1a–c). The minimal binding concentration for antibodies 1A8, 1B2, 1H5, and 1H10 ranged between 0.51 and 1.52 ng/mL for all tested novel H7 HAs of the Eurasian lineage (A/Hunan/02285/2017 (Hunan), A/Guangdong/17SF003/2016 (Guangdong), A/Hong Kong/2014/2017 (Hong Kong)). Binding to novel H7 HAs of the North American lineage (A/feline/New York/16-040082-1/2016 (New York), (A/turkey/Indiana/16-001403-1/2016 (Indiana)) was weaker (Fig. 1d, e). As expected, mAbs 1A8 and 1B2 had low minimal binding concentrations for the North American lineage HAs (4.57–13.72 ng/mL for the feline H7 HA (Fig. 1d) and 1.52 ng/mL for Indiana H7 (Fig. 1e)) indicating a strong binding phenotype. The binding of 1H5 and 1H10 to these isolates was lower, but still detectable (1H5 to feline H7 0.10 µg/mL, 1H10 to feline H7 0.37 µg/mL (Fig. 1d), 1H5 to Indiana H7 0.37 µg/mL, 1H10 to Indiana H7 3.33 µg/mL) (Fig. 1e). These data confirmed that the broadly reactive antibodies raised against the A/Shanghai/1/2013 virus isolate can bind to the H7 HA of novel virus isolates of 2016 and 2017. In fact, the mAbs had similar minimal binding concentrations for novel Eurasian lineage HAs as compared to the Shanghai H7 HA (depicted by the vertical dashed line; Fig. 1a–c). As expected, the binding to the phylogenetically more distant North American lineage HAs was weaker (Fig. 1d, e).

### Characterization of in vitro functionality of mAbs in HI and microneutralization assays

**Fig. 2:**
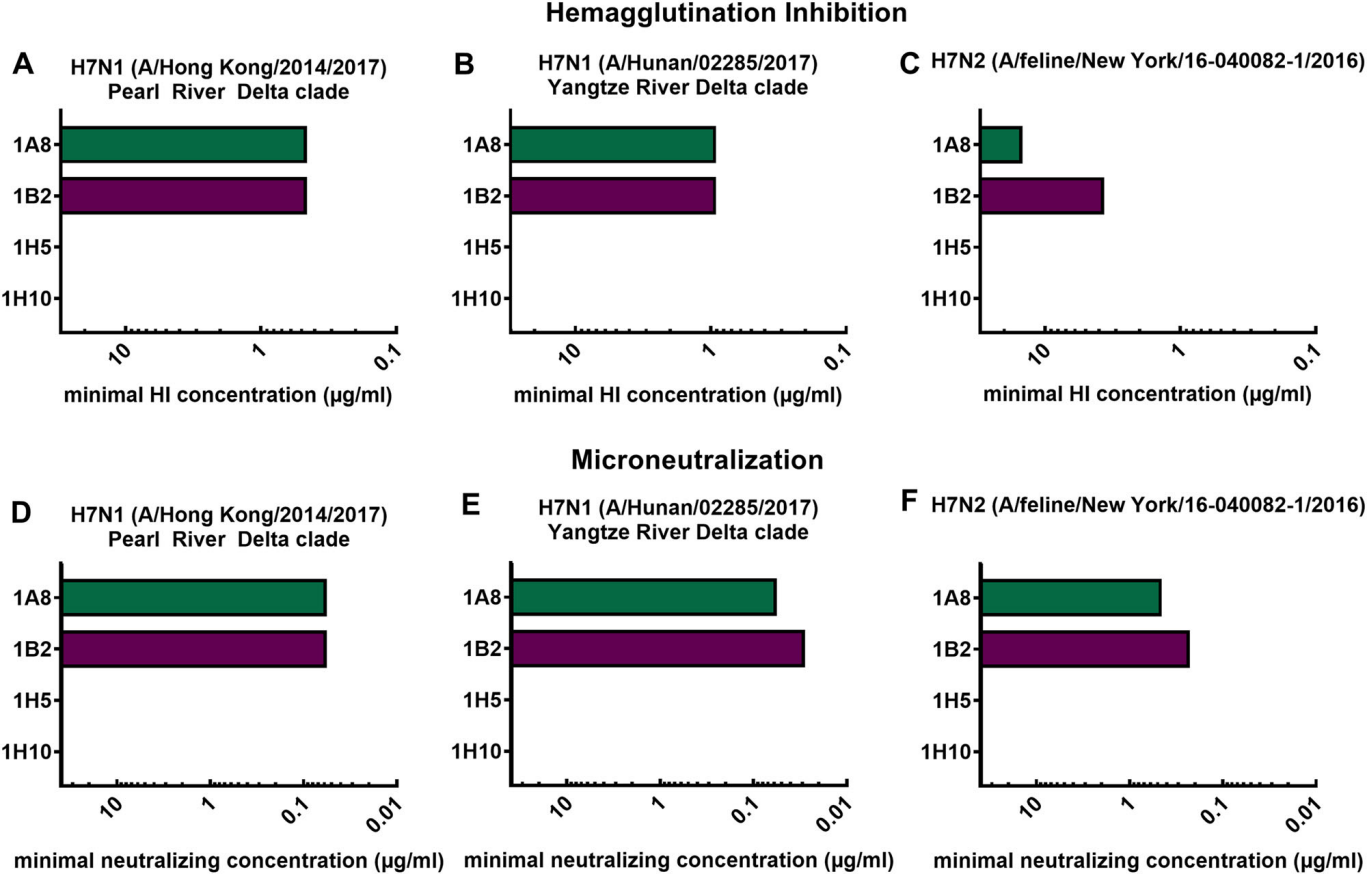
In vitro activity of mAbs in hemagglutination inhibition and microneutralization assays. Minimal hemagglutination inhibition concentrations of mAbs 1A8, 1B2, 1H5, and 1H10 in µg/mL against reassortant viruses. **a** A/Hong Kong/2014/2017 H7N1, (**b**) A/Hunan/02285/2017 H7N1, and (**c**) A/feline/New York/16-040082/2016 H7N2. Minimal neutralizing concentrations of mAbs 1A8, 1B2, 1H5, and 1H10 against the same viruses (**d**–**f**)

In order to characterize the in vitro functionality of the mAbs, we generated (low pathogenic) H7 virus reassortants that express the surface glycoprotein segment (HA) of novel H7 virus isolates by plasmid-based reverse genetic techniques^28^. The HAs of two H7N9 (Hong Kong and Hunan, both low pathogenic variants) virus isolates were each recombined with the six internal segments and the neuraminidase (NA) of laboratory strain PR8, resulting in 7:1 reassortant viruses. For the generation of the New York (A/feline/New York/16-040082-1/2016) reassortant virus, the HA (H7) and NA (N2) were rescued in a PR8 backbone, leading to a PR8-6:2 reassortant. Successful virus rescue and a lack of mutations were confirmed by deep-sequencing. Following the successful virus rescue, they were used to assess antibody functionality. The mAbs 1A8 and 1B2 showed HI activity (Fig. 2a–c) and inhibited novel H7 reassortant viruses at low minimal HI concentrations (1A8 0.47 µg/mL, 1B2 0.47 µg/mL for Hong Kong; 1A8 0.94 µg/mL, 1B2 0.94 µg/mL for Hunan). These concentrations are in the same range as previously reported^27^ for a Shanghai H7N9 (xPR8) virus. The minimal HI concentrations of the mAbs against the feline virus (New York) were higher (1A8 15 µg/mL, 1B2 3.75 µg/mL) (Fig. 2c), and are comparable to an avian H7 virus (A/rhea/North Carolina/39482/1993) of the North American lineage previously tested. As expected from previous results, mAbs 1H5 and 1H10 had no detectable HI activity for the viruses tested (Fig. 2a–c). This is consistent with previously conducted epitope mapping that showed potential binding of these mAbs to the lateral part of the globular head domain in close proximity to the stalk domain^27^. Furthermore, neutralizing activity was assessed using microneutralization assays (Fig. 2d–f). The HI-active antibodies (1A8, 1B2) could also neutralize all three tested viruses (Fig. 2d–f). The minimal neutralizing concentration of 1A8 and 1B2 was 0.06 µg/mL against the Hong Kong virus. Against the Hunan virus isolate, 1A8 neutralized at a concentration of 0.06 µg/mL and 1B2 at 0.03 µg/mL (Fig. 2e). The minimal neutralizing concentration of 1A8 and 1B2 against the New York isolate was 0.47 and 0.23 µg/mL, respectively (Fig. 2f). The non-HI-active mAbs 1H5 and 1H10—as expected—did not show neutralizing potential for the viruses at the tested concentrations (Fig. 2d–f).

### Novel H7 viruses in a PR8 backbone cause morbidity and mortality in the BALB/c mouse model

**Fig. 3:**
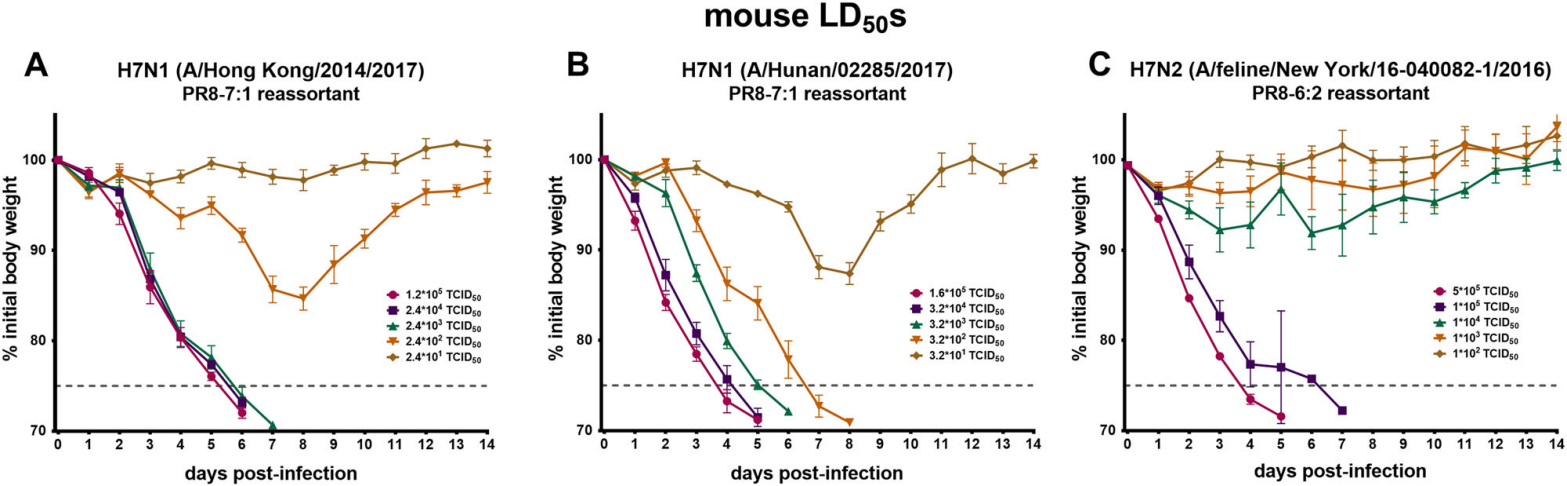
Determination of 50% mouse lethal doses (LD_50_) of PR8-based reassortant H7 viruses. Weight loss curve of 6–8-week-old female BALB/c mice (*n* = 3 per group) infected with different doses of (**a**) H7N1 (A/Hong Kong/2014/2017), (**b**) H7N1 (A/Hunan/02285/2017), and (**c**) H7N2 (A/feline/New York/16-040082/2016). The error bar indicate the standard error of the mean. The weight loss and survival were monitored for 14 days. The dashed gray line represents 75% initial body weight, which was defined as the humane endpoint

Subsequently, to prepare for in vivo mAb protection studies, we tested whether the rescued H7 viruses were able to infect and replicate in mice. Female BALB/c mice were intranasally infected with the H7 PR8 reassortants (PR8-7:1 or PR8-6:2) and weight loss was monitored daily for 14 days (Fig. 3a–c). All three H7 viruses: the YRD clade (Hunan), the PRD clade (Hong Kong), and the New York (PR8-6:2) virus conferred morbidity and mortality (Fig. 3). The murine lethal dose 50 (LD_50_) for the H7 Hong Kong isolate was reached at a viral input of 250 times the tissue culture infection dose 50 (TCID_50_) per 50 µL. For the Hunan isolate the LD_50_ was at 253 × TCID_50_s/50 µL. The LD_50_ value of the New York feline virus was 3.16 × 10^4^ × TCID_50_/50 µL. The mice dropped below 75% initial body weight starting from days 4 to 7 (Hong Kong, New York) or day 8 (Hunan) and had to be euthanized. These data indicate that all three reassortant H7 viruses infected mice, resulting in morbidity and mortality and could be used for subsequent experiments.

### Cross-reactive H7 mAbs confer protection from lethal virus challenge in prophylactic and therapeutic settings in the mouse model

**Fig. 4:**
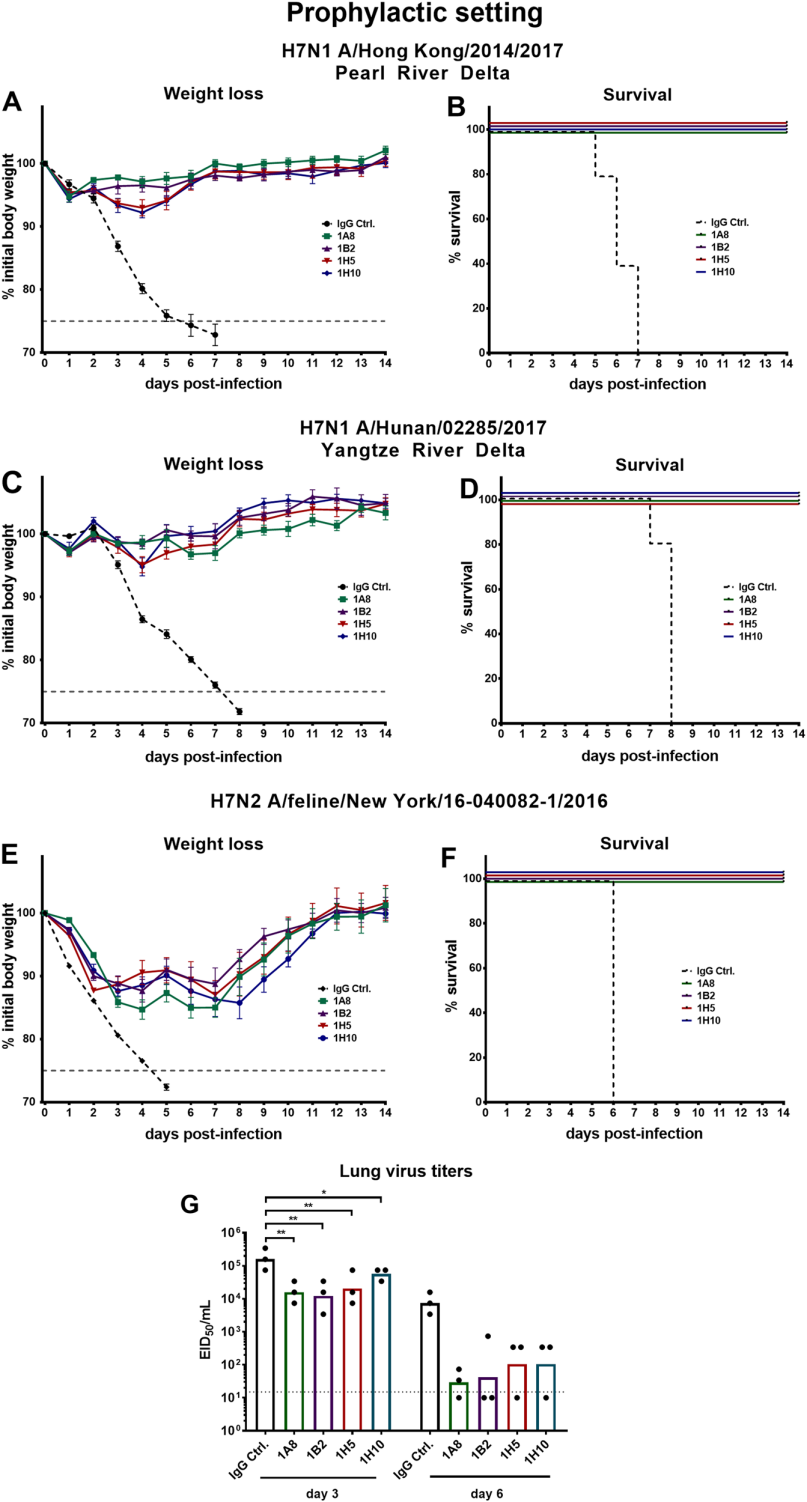
Protective efficacy of mAbs in a prophylactic setting against lethal virus challenge in the mouse model. **a**, **c**, **e** show weight loss curves of animals pretreated (*n* = 5 per group) with monoclonal antibodies at a concentration of 1 mg/kg and challenged with H7N1 A/Hong Kong/2014/2017, H7N1 A/Hunan/02285/2017, or H7N2 A/feline/New York/16-040082/2016 reassortant viruses 2 h post mAb transfer. The error bars represent the standard error of the mean. The dashed black line represents treatment with a negative control IgG and the dashed gray line represents 75% weight loss. **b**, **d**, **f** Survival graphs showing percent survival in the different groups used in the prophylactic mouse challenge model. **g** The lung viral titers on days 3 and 6 post infection are shown as EID_50_/mL for IgG control and mAbs 1A8, 1B2, 1H5, and 1H10 (*n* = 3 per group). The dotted line represents the limit of detection (10 × EID_50_/mL). Lung virus titers of the IgG control were compared for the same day (3 or 6) in a one-way ANOVA with a Sidak post test for multiple comparison. Significance is indicated as follows: *P* > 0.05; *P ≤ 0.05; **P ≤ 0.01

After assessing inhibition and neutralization activity of the antibodies in vitro the novel H7 virus reassortants were used to investigate whether the four broadly reactive H7 mAbs confer protection in vivo. The protective effect of the mAbs was tested both in a prophylactic setting and by therapeutic administration of mAbs post infection. For the prophylactic treatment female 6–8-week-old BALB/c mice received 1 mg/kg of a mAb and were challenged with 5 × LD_50_ (Hunan and Hong Kong). Both the neutralizing mAbs 1A8 and 1B2 and the non-neutralizing, non-HI-active antibodies 1H5 and 1H10 fully protected against lethal challenge with H7N1 Hong Kong and Hunan reassortant viruses (Fig. 4a–d). The negative control mice lost weight and succumbed to infection on days 5 to 7 (Hong Kong) and on days 7 to 8 (Hunan) post infection (Fig. 4a–d). To test the protective efficacy of the mAbs against a North American lineage virus, mice were challenged with 2 × LD_50_ of feline PR8 reassortant virus. The viral input dose needed to induce mortality in mice for the New York isolate was substantial (LD_50_ of 3.16 × 10^4^ × TCID_50_/50 µL; Fig. 3c). To avoid losing sensitivity and challenging the mice with too much virus (which could lead to unwanted morbidity early after infection due to innate immune responses triggered by massive virus input), a lower viral input dose (2 × LD_50_ instead of 5 × LD_50_) was selected. All mice that received broadly reactive H7 antibodies were fully protected from lethal challenge but showed morbidity (10–15% weight loss) before recovering at day 8 (Fig. 4e). The negative control mice succumbed to infection on day 6. (Fig. 4f). As described above, mAb-treated mice challenged with Eurasian lineage H7 viruses showed no morbidity, whereas the mAb-treated mice challenged with the North American lineage feline H7 virus showed weight loss. Therefore, the reduction of lung virus titers was assessed for the New York virus to determine the mAbs’ ability to clear infection and to investigate if there are differences between neutralizing and non-neutralizing mAbs. Mice were given 5 mg/kg mAbs (1A8, 1B2, 1H5, 1H10, or immunoglobulin G (IgG) control). After 2 h, the mice were infected with 0.1 × LD_50_. This low dose was selected to avoid that the (control) mice succumb to infection before day 3 or more importantly day 6 and to allow for a more sensitive readout. To assess lung titers, the lungs were harvested at 3 or 6 days post infection and the lung titers determined in the form of egg infectious dose 50s (EID_50_s). All H7-specific mAbs reduced viral lung titers on days 3 and 6 (Fig. 4g) as compared to a control IgG. It has to be noted that 1A8 and 1B2 had lower minimal binding concentrations than 1H5 and 1H10 as demonstrated before (indicating stronger binding), which might explain the subtle differences observed in lung virus reduction. However, non-neutralizing, non-HI-active antibodies 1H5 and 1H10 significantly reduced viral lung titers on day 3 and reduced the titers on day 6 as well.

**Fig. 5:**
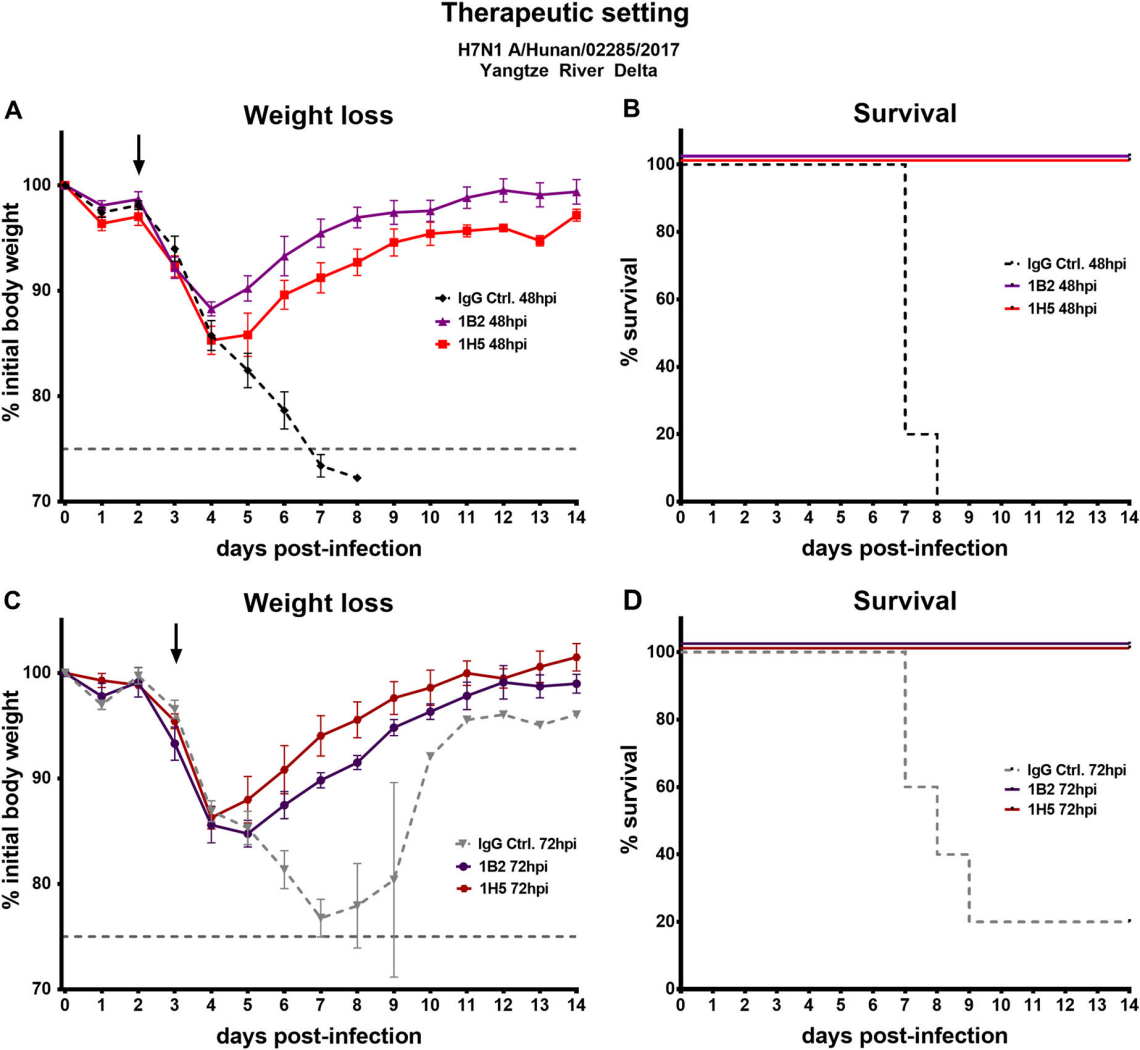
Protective therapeutic efficacy of broadly reactive H7 antibodies in a lethal mouse challenge model. **a**, **c** Weight loss curves of animals receiving antibody 1A8, 1B2, 1H5, or 1H10 at a concentration of 5 mg/kg 48 or 72 h post infection. The dashed lines represent negative control groups. The dashed vertical gray line represents 75% weight loss. The black arrows indicate when mAbs were given. **c**, **d** Percent survival of mice receiving therapeutic mAb treatment in different groups

To determine if the mAbs were also protective in a therapeutic setting, mice were challenged with 5 × LD_50_ of Hunan virus (YRD). Viruses of the YRD clade were predominantly detected in individuals with H7N9 infections within the fifth wave of the H7N9 epidemics and the Hunan virus was selected for therapeutic testing for this reason^9^. After 48 or 72 h 5 mg/kg 1B2 or 1H5 mAb were administered intraperitoneally. Here, one neutralizing (1B2) and one non-neutralizing (1H5) antibody was selected. The selection was based on ELISA data that showed slightly stronger binding of neutralizing mAb 1B2 over 1A8 and non-neutralizing 1H5 over 1H10 to novel H7 HAs, and to reduce the number of mice used. The mice that received mAbs, either 48 or 72 h post infection, recovered shortly after administration, gained weight, and were fully protected from lethal challenge (Fig. 5a–d). The IgG control mice succumbed to infection on days 7 to 9, except for one mouse in the 72 h post infection IgG control group that survived the challenge. These data show that all four mAbs were protective in vivo, reduced lung virus titers, and could be applied as prophylactics and/or therapeutics.

### mAbs 1A8 and 1B2 crossreact to the HA of emerging H7N9 viruses despite changes in their target antigenic site A

**Fig. 6:**
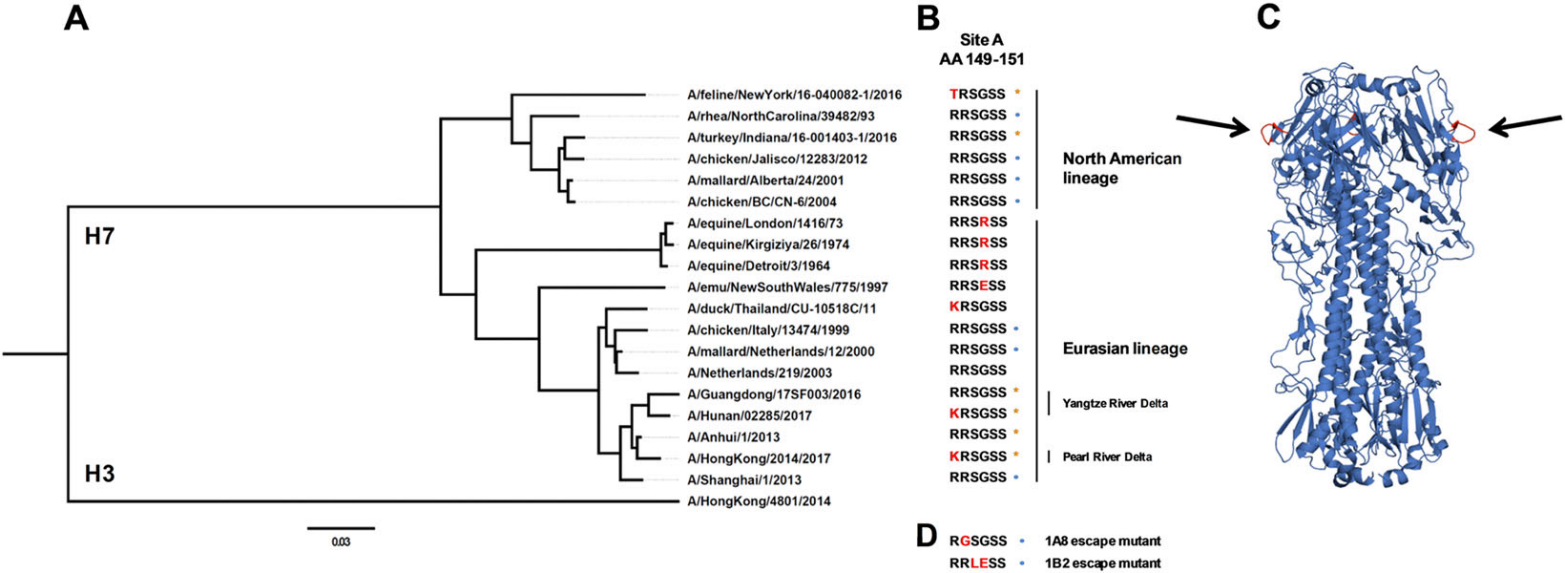
Phylogenetic tree of H7 HAs, antigenic site A, and structure of influenza A H7 HA. **a** Phylogenetic tree based on the amino acid sequence of H7 HAs. The amino acid sequence of H3 (A/Hong Kong/4801/2014) was used as an outgroup. The scale bar at the bottom shows a 3% difference in amino acid identity. **b** Amino acids of H7 HA at position 149 to 151 (antigenic site A; H3 numbering) are shown. Red letters represent differences to A/Anhui/1/2013 (and A/Shanghai/1/2013). Orange stars represent H7 HAs used in this study and blue circles H7 HAs tested in the previous report^27^. The different lineages (North American vs. Eurasian) and clades (Pearl River Delta vs. Yangtze River Delta) are indicated. **c** Graphic representation of the crystal structure of the A/Anhui/1/2013 H7 HA trimer. Black arrows are pointing at the antigenic site A shown in red. The structure is based on PDB # 4R8W^48^. **d** Mutations of antigenic site A that resulted in loss of binding of mAbs 1A8 and 1B2 as determined by escape mutagenesis (as previously reported)

In our previous report^27^, epitope analysis showed that neutralizing mAbs 1A8 and 1B2 target an epitope that overlaps antigenic site A of H7 HA (Fig. 6c). To further investigate the conservation of this critical H7 epitope, we generated a phylogenetic tree based on the amino acid sequences of H7 HAs, which showed high divergence between different isolates (Fig. 6a). A stark contrast could be observed between North American and Eurasian lineage H7 HA sequences. Nevertheless, a common, conserved motive found in all H7 HA sequences was antigenic site A. As previously reported^27^, the amino acid sequence of antigenic site A found in Eurasian lineage H7 HAs was RRSGSS in about 83% of isolates, and 49% in North American isolates. The second major sequence found in North American isolates was TRSGSS (38% of isolates). Previously generated escape mutants^27^ indicated that the mAbs bound to the sequence RRSGSS (antigenic site A), and mutations in amino acids within that site (Fig. 6d) led to a loss or reduction of binding. The HA of the Hunan and Hong Kong virus isolates used in this study had a mutation in antigenic site A in position 148 (according to H3 HA numbering) changing arginine (R) to lysine (K) (Fig. 6c). As shown by ELISA, in vitro, and in vivo experiments, a mutation in this position had no detectable influence on mAb binding and function. It has to be noted that R and K are similar amino acids and that a change to another amino acid might have a different impact. The HA of the New York virus isolate had a different amino acid in position 148 (threonine (T) instead of arginine (R)). This variant of antigenic site A is the second most commonly found sequence in North American isolates as mentioned above. Again, the antibodies did bind to the HA and did not lose function, but showed increased minimal binding concentrations as compared to Eurasian lineage HAs. This indicates that the usually highly conserved antigenic site A was changing. So far, mAbs raised against the Shanghai HA isolate were still reactive and functional. It is however unclear what might happen if other amino acids mutate, like those that led to an escape in previous experiments^27^ or if other positions within site A change.

## Discussion

Influenza viruses of the H7 subtype pose a pandemic threat. Zoonotic H7 viruses have shown to possess two out of three major factors that drive the pandemic potential of an influenza virus including their ability to cause human disease and the fact that immunity of the population to these virus strains is very low to non-existent^5^. So far, no substantial transmission of H7 influenza A viruses (IAVs) between humans has been observed, which would be the third major requirement for a virus to cause widespread human disease and potentially become pandemic. Reassortment of zoonotic H7 viruses with seasonal human IAV strains could, in theory, facilitate the generation of viruses with high transmission potential^15,29^. To date, no reassortment events have been reported and incompatibilities at the RNA or protein level, called segment mismatch, might potentially prevent certain human IAV strains from easily recombining with zoonotic influenza virus strains^30^. Nevertheless, it is important to better understand the antigenicity of H7 viruses^31^ and to establish potential therapeutics for pandemic preparedness^32^.

In the present study, we used mouse mAbs as a tool to characterize the conservation of epitopes between novel H7 viruses. H7N9 viruses in China are evolving, but at least two epitopes of the H7 HA are unchanged and can still be targeted by broadly reactive antibodies. These findings are consistent with the high sequence identity (95–98%) of A/Shanghai/1/2013 and the novel Asian lineage H7 HAs. Interestingly, the mAbs also bound to the more diverged HA of a feline H7N2 virus and the H7 HA of a highly pathogenic avian H7N8 isolate. The isolates of the North American lineage tested were phylogenetically very distinct from the H7 A/Shanghai/1/2013 HA used for the generation of the mAbs and antibody binding in ELISA to North American isolate HAs was weaker. Importantly, we showed that cross-reactive mouse mAbs can still be used as effective prophylactic or therapeutic agents in animal challenge experiments. This suggests that humanized or human mAbs targeting the same or similar epitopes could be developed for potential human application. It typically takes about at least 6 months from the preparation of a seed virus strain until a vaccine can be shipped and administered^14^. In case of a pandemic outbreak, the population is vulnerable to infection and disease during this time frame^33^. A readily available cocktail of broadly reactive anti-H7 mAbs could help to bridge this gap and serve as an anti-viral agent while matched vaccines are being developed^34^.

Previously generated neutralizing as well as non-neutralizing, non-HI-active H7 HA-reactive mAbs conferred protection in mouse passive transfer experiments. Specifically, non-HI-active antibodies that confer protection by Fc-mediated effector functions, like antibody-dependent cell-mediated cytotoxicity (ADCC), antibody-dependent cellular phagocytosis (ADCP), and complement-dependent lysis (CDL), are of recent interest^23,24,26,35^. These mAbs cannot readily be detected by classical HI assays. For instance, it has been demonstrated that the non-HI-active, non-neutralizing mAbs 1H5-mediated and 1H10-mediated ADCC activity as measured in a bioreporter assay^27^.

The North American and Eurasian lineage H7 HAs are genetically highly divergent^36^. However, antigenic site A is conserved, present on HAs of both lineages and a potentially important protective epitope^37^. As previously shown, antigenic site A is also recognized and targeted by the human immune system after vaccination with an H7N9 LAIV and IIV boost regimens^24,38^. The mAbs 1A8 and 1B2 that target antigenic site A were neutralizing, which allowed for the generation of escape mutants. Sequence analysis of the escape mutants showed that a change of amino acid at position 2 (H3 numbering position 149) or 3 and 4 (position 150, 151) of antigenic site A led to an escape. Another group showed the same amino acid change (R149G), leading to a loss of neutralization activity^37^. As demonstrated, naturally occurring mutations of amino acids at position 148 did not influence binding, function, or protective efficacy of mAbs raised against the wild-type sequence of the 2013 H7 HA (Shanghai).

In conclusion, we showed that antibodies raised against the H7 HA from a 2013 strain bound to H7 HAs of novel zoonotic viruses isolated in 2016 and 2017. These findings suggest that while the H7 HAs evolve; highly conserved epitopes on the H7 HA are still maintained, possibly because of a lack of antigenic pressure on the site in the avian host species. Ideally, the human immune response could be directed against these epitopes by vaccination to elicit cross-protection against several different H7 virus strains. Based on our data, it is likely that stockpiled vaccines based on the Shanghai virus strain could confer at least some protection against divergent, novel H7 viruses. Indeed, we recently showed that antibodies induced by vaccination with recombinant H7 HA from the A/Anhui/1/2013 strain react to the HAs of emerging H7 viruses^19^. Nevertheless, assessment of the antigenicity of emerging strains is of utmost importance to detect mismatches of vaccine strain and novel virus strains. Our findings help inform the development of pre-pandemic influenza vaccines and offer tools to characterize and evaluate new zoonotic or human IAV H7 virus strains and candidate vaccine viruses.

## Material and methods

### Cells, viruses, and proteins

Madin Darby canine kidney (MDCK) cells were grown and maintained in Dulbecco’s modified Eagle’s medium (DMEM; Gibco) containing penicillin–streptomycin antibiotics mix (100 U/mL of penicillin, 100 µg/mL streptomycin; Gibco) and fetal bovine serum (10%; HyClone), resulting in complete DMEM (cDMEM). BTI-TN5B1-4 (*Trichoplusia ni*) cells were grown in serum-free SFX medium (HyClone) supplemented with antibiotics (100 U/mL of penicillin, 100 µg/mL streptomycin; Gibco). Human embryonic kidney cells (293T) were grown in cDMEM. The H7 low pathogenic virus reassortants were generated by plasmid-based reverse genetic techniques as described previously^28^. Briefly, the H7 HA of A/Hunan/02285/2017 and A/Hong Kong/2014/2017 were combined, respectively, with seven genomic segments of A/Puerto Rico/8/1934 (PR8) resulting in 7:1 reassortants. The H7 and N2 of the A/feline/New York/16-040082/2016 were combined with the six internal segments of PR8 producing a 6:2 reassortant. The PR8 backbone was chosen because it is attenuated in humans (but not in mice), does not confer a transmittable phenotype and is generally regarded as safe in humans, poultry, and ferrets^39–43^. The HA cDNAs were synthetically generated (Thermo Fisher), and all recombinant viruses produced by reverse genetics were sequenced to confirm genotype. None of the rescued viruses contained a polybasic cleavage site in their HA sequence. The viruses were grown in 8–10-day-old embryonated chicken eggs (Charles River Laboratories) for 48 h at 37 °C and the allantoic fluid harvested. The recombinant proteins, including H7 from A/Hunan/02285/2017, A/Hong Kong/2014/2017, A/feline/New York/16-040082/2016, A/turkey/Indiana/16-001403-1/2016, and A/Guangdong/17SF003/2016 were expressed in the baculovirus expression system as described previously^44,45^. To increase recombinant protein stability, the polybasic cleavage sites of the HA of the highly pathogenic isolates A/turkey/Indiana/16-001403-1/2016 and A/Guangdong/17SF003/2016 were removed. The resulting sequences have regular monobasic low pathogenic avian influenza H7 cleavage sites.

### mAb generation and purification

The H7-specific mAbs 1A8, 1B2, 1H5, and 1H10 were generated by hybridoma technology as previously described^27^. The antibodies were purified from 800 mL culture supernatant via sepharose G columns using a standard protocol^46^.

### Enzyme-linked immunosorbent assay

Ninety six-well microtiter plates (Thermo Fisher) were coated with 50 µL recombinant protein at a concentration of 2 µg/mL in coating buffer (KPL) overnight at 4 °C. The next day, 220 µL blocking solution (phosphate-buffered saline (PBS; Gibco) supplemented with 0.1% Tween-20 (T-PBS; Fisher Scientific), 0.5% milk powder (AmericanBio), and 3% goat serum (Life Technologies)) were added to all wells of the microtiter plates and incubated for 1 h at room temperature. mAbs were diluted to a starting concentration of 10 µg/mL, serially diluted 1:3, and incubated for 2 h at room temperature. The microtiter plates were washed three times with T-PBS and 50 µL anti-mouse IgG (whole molecule) peroxidase antibody (produced in rabbit; Sigma, #A9044) diluted 1:3000 in blocking solution was added to all wells and incubated for 1 h at room temperature. The microtiter 96-well plates were washed four times with T-PBS and were developed with 100 µL/well SigmaFast *o*-phenylenediamine dihydrochloride (Sigma). After 10 min the reaction was stopped with 50 µL 3 M hydrochloric acid (Thermo Fisher) and the plates were read at 490 nm with a microtiter plate reader (BioTek). The data were analyzed in Microsoft Excel and GraphPad Prism. The cutoff value was defined as the average of all blank wells plus three times the standard deviation of the blank wells and the area under curve values were calculated.

### HI assay

The mAbs were diluted to an initial concentration of 30 µg/mL in PBS and serially diluted 1:3 in V-bottom plates 96-well plates (Thermo Fisher). The viruses were diluted to 8 hemagglutination units/50 µL in PBS and 25 µL of virus was added to the serially diluted 25 µL/well of mAb dilutions. The plates were incubated at room temperature for 30 min on a shaker. Chicken red blood cells (RBCs; Lampire Biologicals) were diluted to a concentration of 0.5% in PBS and 50 µL was added to each well of the V-bottom plates. The plates were incubated at 4 °C until the formation of red pellets on the bottom of the wells of the negative control wells were visible (45–60 min). The minimal HI concentration was defined as the last dilution (concentration of antibody) in which hemagglutination does not occur. The results were analyzed in Microsoft Excel and GraphPad Prism 7.

### Microneutralization assay

MDCK cells (100 µL/well) were seeded at a concentration of 2 × 10^5^ cells/mL in 96-well cell culture plates (Sigma) and incubated at 37 °C for 12 h. The mAbs were diluted to a starting concentration of 30 µg/mL in PBS and serially diluted 1:2 in UltraMDCK media (Lonza) supplemented with tosyl phenylalanyl chloromethyl ketone-treated trypsin (infection media; Sigma) at a concentration of 1 µg/mL, in 96-well cell culture plates (Sigma). The viruses were diluted to a concentration of 100 × TCID_50_/50 µL (A/Hunan/02285/2017 (Hunan), A/feline/New York/16-040082/2016 (New York), A/Hong Kong/2014/2017 (Hong Kong)) in infection medium. Next, 60 µL of virus dilution was incubated with 60 µL of mAb serial dilution and incubated on the shaker at room temperature for 1 h. The plates were incubated at 33 °C for 48h  (New York) or 72 h (Hunan, Hong Kong). The readout was performed by the means of classical hemagglutination assay. This readout was chosen because it is more objective than assessment of cytopathic effects but easier to perform than staining for virus antigen (e.g., for nucleoprotein). In brief, chicken RBCs (Lampire Biological Laboratories) was diluted to a concentration of 0.5% in PBS and added to 50 µL of cell supernatant in v-bottom plates (Corning). After 45–60 min the plates were scanned and the results analyzed in Microsoft Excel and GraphPad Prism 7.

### Passive transfer experiments in mice

Passive transfer experiments were performed to test prophylactic and therapeutic efficacy of the mAbs. For the prophylactic setting, 6–8-week-old female BALB/c mice (*n* = 5 mice/group) were given 150 µL mAb 1A8, 1B2, 1H5, or 1H10, at a concentration of 1 mg/kg intraperitoneally. The negative control mice received 150 µL of irrelevant IgG (anti-Ebola virus glycoprotein mAb 2E5^47^) control at a concentration of 1 mg/kg. Two hours post transfer, the mice were anesthetized with a ketamine–xylazine–water mixture (0.15 mg ketamine/kg of body weight and 0.03 mg/kg xylazine; 100 µl intraperitoneally) and challenged intranasally with 5 × LD_50_ (1265 TCID_50_/50 µL H7N1; A/Hunan/02285/2017), 5 × LD_50_ (1250 TCID_50_/50 µL H7N1; A/Hong Kong/2014/2017), or 2 × LD_50_ (6.32 × 10^4^ TCID_50_/50 µL H7N2; A/feline/New York/16-040082/2016). Blood was drawn and analyzed by ELISA as previously described to confirm successful antibody transfer^26^. The weight loss was monitored daily for 14 days. The humane endpoint was defined as a loss of 25% of the day 0 weight. To test the therapeutic effect of the antibodies mice were infected with 5 × LD_50_. MAbs 1H5, 1B2 and negative control mAb 2E5 (anti-Ebola virus glycoprotein mAb) were administered 48 or 72 h post infection at a concentration of 5 mg/kg. Weight loss was monitored for 14 days and mice that lost 25% or more of their initial body weight were euthanized according to institutional guidelines. For the determination of reduction of lung viral titers, mice were infected with 0.1 × LD_50_ (3.16 × 10^3^ TCID_50_/50 µL) H7N2 A/feline/New York/16-040082/2016 (xPR8) virus. Two hours prior infection, mAbs 1A8, 1B2, 1H5, and 1H10, at a concentration of 5 mg/kg and a negative IgG control (anti-Ebola virus glycoprotein), were transferred via intraperitoneal injection. At day 3 (*n* = 3 mice/mAb) and day 6 (*n* = 3 mice/mAb) post infection, lungs were harvested and homogenized using a BeadBlaster24 (Benchmark). Lung virus titers were assessed by injecting dilutions (1:5, 1:50, 1:500, 1:5000, 1:50,000, 1:500,000) of lung homogenate into 8-day-old embryonated chicken eggs (Charles River Laboratories) and incubation for 48 h at 37 °C. The eggs were harvested and presence or absence of virus determined by classical HA readout as described before^19^. The EID_50_ was calculated in Microsoft Excel and GraphPad Prism 7.

### HA sequences

The sequences for the analysis of antigenic site A of the H7 HA were downloaded from the Influenza Resource Database (www.fludb.org) and the Global Initiative on Sharing Avian Influenza Data (www.gisaid.org).

### Statistical analysis

Statistical analysis was performed in GraphPad Prism 7. Data are shown as geometric means. Differences in lung virus titers were compared in a one-way analysis of variance (ANOVA) with a Sidak post test for multiple comparisons. The sequences for the phylogenetic tree were assembled in Clustal Omega and visualized in FigTree.

### Data availability

The data that support the findings of this study are available from the corresponding author upon request.
